# Investigation of cryotherapy for pain relief after arthroscopic shoulder surgery

**DOI:** 10.1186/s13018-022-03404-x

**Published:** 2022-12-20

**Authors:** Rinko Uchida, Amy Hombu, Yasuyuki Ishida, Makoto Nagasawa, Etsuo Chosa

**Affiliations:** 1 School of Nursing, Faculty of Medicine, University of Miyazaki, 5200 Kihara Kiyotake-Cho, Miyazaki-City, Miyazaki 889-1692 Japan; 2 Center for Language and Cultural Studies, University of Miyazaki, Miyazaki, Japan; 3 Ishida Orthopaedic Clinic, Miyazaki, Japan; 4 Department of Orthopaedic Surgery, Faculty of Medicine, University of Miyazaki, Miyazaki, Japan

**Keywords:** Cryotherapy, Pain relief, Shoulder arthroscopy, Rotator cuff repair, Postoperative, Cooling temperature, Duration of cooling

## Abstract

**Background:**

Recently, cryotherapy has become a common practice for postoperative pain management. The current accepted practice in Japan is the use of cryotherapy at 5 °C after arthroscopic shoulder surgery. However, this therapy has been reported to be highly intense because the sustained low temperature causes discomfort for patients. The optimum temperature and duration of cooling required for comfortable and effective cryotherapy after arthroscopic shoulder surgery were investigated.

**Methods:**

Because pain levels might differ depending on the condition, we selected 52 patients with rotator cuff injuries, which were the most common disorders indicated for arthroscopic shoulder surgery. Patients were treated with cryotherapy at 5 °C or 10 °C for 16 h or 24 h. The pain level was determined using the visual analogue scale, and deep shoulder joint temperatures were recorded at different time points for analysis.

**Results:**

Pain after arthroscopic shoulder surgery was found to be related to the presence of a brachial plexus block using the interscalene approach during surgical anesthesia. To obtain effective analgesia with cryotherapy, the cooling temperature and duration of cryotherapy had to be changed based on the presence or absence of the brachial plexus block. Patients who received brachial plexus blocks had the lowest recorded pain scores after receiving cryotherapy at 5 °C for 24 h after surgery. Patients who did not receive the block had the lowest recorded pain scores when receiving cryotherapy at either 5 °C for 16 h or 10 °C for 24 h.

**Conclusions:**

Using universal cryotherapy intensity and duration settings regardless of the use of other interventions is likely to unintentionally increase postoperative pain levels. This study revealed that cryotherapy at 5 °C for 24 h was optimal for patients who received an anesthesia block and at 5 °C for 16 h or at 10 °C for 24 h for those who did not receive the anesthesia block. These results can be used as a reference for setting the temperature and duration of cryotherapy after arthroscopic shoulder surgery.

## Background

Cryotherapy has been popular in the field of sports medicine since the 1950s, and research of its methodology and effectiveness in clinical practice has been conducted since the 1980s. Initially, intermittent cooling was performed using an ice bag, but sustained cooling at a constant temperature was considered more effective. Even though the equipment for icing systems was introduced in the late 1990s, the effective cooling temperature and duration remain unclear.

The recovery period after arthroscopic shoulder surgery invariably involves a certain degree of pain for patients [1–4]. Postoperative pain experienced by patients who have undergone arthroscopic shoulder surgery was found to be related to the presence of a brachial plexus block using the interscapular approach (anesthesia block) during surgical anesthesia [5–17]. Postoperative recovery varies greatly for each person and is also related to the use of pain reduction techniques, prompt initiation of rehabilitation, promotion of blood circulation, prevention of muscle contracture, and restoration of the range of motion in the shoulder joint [3, 18–26].

Cryotherapy is the cornerstone of postoperative management [23, 27–29] and is used to reduce the temperature of the affected area, mainly the skin surface, and decrease cell metabolism and blood flow, thereby reducing swelling, pain, muscle spasms, and inflammation. However, cryotherapy also involves the risk of circulatory disturbance caused by “overcooling” and potential adverse effects such as suppression of wound healing attributable to the low temperature [23, 30, 31]. Therefore, the specifics regarding the optimal application of cryotherapy as a postoperative technique for pain reduction are critical to providing the greatest benefits and least burden to patients.

Because of the short history of cryotherapy use after arthroscopic shoulder surgery in Japan (< 30 years), according to the Japan Shoulder Society, each medical institution using cryotherapy provides different intensities and durations of cooling. The physiological effects of cryotherapy were studied by Knight et al. [30], and there is a large body of the literature about cryotherapy [23, 28, 29, 31]. However, there is insufficient knowledge of its effects on inflammation, relevant methodological approaches, indications, and duration of use [23, 30]. Therefore, to allow for comfortable, effective, and efficient cryotherapy after arthroscopic shoulder surgery, we investigated the optimal cooling temperatures and durations.

## Methods

Cryotherapy was performed as part of postoperative management between March 30, 2017, and November 15, 2018, for patients of 16 years or older who underwent arthroscopic shoulder surgery. This was a prospective study. At the beginning of the study, we considered investigating all cases of arthroscopic shoulder surgery at our hospital (approximately 60 surgical cases per year). Because the cooling temperature and duration of cooling considered most effective for cryotherapy have not yet been clarified, the patients were randomly divided into the following four groups: 5 °C for 24 h; 5 °C for 16 h; 10 °C for 24 h; and 10 °C for 16 h. Due to the shortage of measuring equipment, we were able to collect the data of only one patient at a time. Patients were randomly divided into four groups based on several conditions. If the patient underwent surgery during the morning, then the temperature was set at 5 °C for 24 h. When the number of patients reached approximately 15, the temperature was shifted to 10 °C for 24 h. If the patient underwent surgery during the afternoon, then the temperature was set at 5 °C for 16 h. When the number of patients reached approximately 15, the temperature was shifted to 10 °C for 16 h. The duration was chosen so that cooling would not limit the patient’s activities the next day or affect early ambulation. At the beginning of this study, there were 66 patients. However, complete information was only available for 64 patients: 52 had rotator cuff injuries, nine had repetitive shoulder dislocations, and three had other conditions.

Because pain might differ based on the condition, we focused on the 52 patients with rotator cuff injuries, which were the most common disorders in this study. All cases of rotator cuff injuries were included in the study. The cause of injury, such as work-related injuries, car accidents, reoperations, or complications of underlying diseases such as diabetes mellitus were not considered. In this study, there were no cases of reoperation, diabetes, or other underlying complications. Therefore, there were no exclusion criteria for patient selection. After this adjustment, the included patients were 42 years or older. Fifteen patients were subjected to 5 °C for 24 h, 13 patients to 5 °C for 16 h, nine patients to 10 °C for 24 h, and 15 patients to 10 °C for 16 h.

Whether a brachial plexus block using the interscalene approach (anesthesia block) comprising ropivacaine hydrochloride hydrate was performed depended on the anesthesiologist on duty at the time of surgery. If an anesthesia block was used, then it was followed by inhalation anesthesia with sevoflurane. If no anesthesia block was used, then sevoflurane inhalation anesthesia was administered.

Cryotherapy provided effective and accessible pain relief for patients who underwent arthroscopic shoulder surgery [23, 29–31] and has been an accepted practice in Japan. Therefore, this study was conducted under the assumption that cooling therapy is effective. The goal was to determine the optimal temperature and duration of cryotherapy. Because of the number of patients who undergo surgery annually, it was necessary to prioritize data collection. Therefore, to ensure that patients who did not require cooling therapy were not subjected to it, no control group was included in this study.

The lowest temperature that could be set on the cooling system in the orthopedic surgery ward was 4 °C. However, 5 °C was chosen for the cooling temperature because temperatures below 5 °C can lead to nerve damage [30]. Additionally, a previous study reported that 5 °C was more effective than cold water (approximately 13 °C) [32]. Therefore, we conducted this study using the conventional temperatures (5 °C and 10 °C), which are colder than that of the water used for alternative cryotherapy methods.

Cryotherapy was performed immediately after returning to the room in the postoperative ward. The Icing system CF-4000 was used after covering the affected area with a cooling pad and immobilizing it so that it would not be compressed, could be observed, and could be removed as needed. To ensure consistency, fixing of the cooling pad was performed for all patients by the same nurse (the principal investigator of this study), and placement of the deep temperature probe and fixing of the gauze was performed for all patients by the same physician who performed the surgery (one of the co-authors of this study).

The following outcomes were measured and compared: body temperature; deep shoulder joint temperature; objective pain as indicated by the pain level based on the PAINVISION PS-2100 instrument used in clinical practice in Japan; subjective pain as indicated by the visual analogue scale (VAS) score; and C-reactive protein (CRP) level. To determine the deep shoulder joint temperature, the Coretemp CM-210 probe was placed at one location in the acromioclavicular joint region on the affected side by the surgeon at the end of surgery. The healthy side was measured by the nurse and surgeon upon return to the room in the postoperative ward. The temperature-sensitive part of the probe tip, which is bonded to the skin, would generate high heat and cause a burn if the Coretemp was maintained on; therefore, the temperature was read by turning it on at the measurement time and turning it off at the end of the measurement. The deep shoulder joint temperatures were measured immediately after the patient returned to the room in the postoperative ward and at 2, 6, 12, 16, 18, and 24 h postoperatively. The recorded pain level was determined using the average of two measurements obtained with the PAINVISION PS-2100 quantitative pain analysis device. The pain level and VAS score were recorded immediately, 2 h later, and every 6 h until 48 h after returning to the room in the postoperative ward. The CRP level was measured during the immediate postoperative period and 1, 4, and 7 days after surgery.

Data analysis was performed using IBM SPSS Statistics version 25. The nonparametric Wilcoxon rank sum test was performed to compare the pain level. Student’s t test was performed for all other comparisons. Pearson’s correlation analysis was also performed to evaluate relationships.

## Results

The mean age of the patients included in this study was 62.9 years (± 9.2 years), and 41 patients (78.8%) were men and 11 (21.2%) were women. The mean operative time was 131.6 min (± 38.4 min). Seven patients (13.5%) underwent surgery for one tendon, 34 patients (65.4%) underwent surgery for two tendons, and 11 patients (21.2%) underwent surgery for three tendons. Twenty-eight patients (53.8%) underwent tenotomy. Table 1 shows the background characteristics of the patients in all four groups.

**Table 1:** Background characteristics of the patients

	5 °C								10 °C							
	24-h group *n *= 15				16-h group *n *= 13				24-h group *n *= 9				16-h group *n *= 15			
Sex (*n*)	Male		Female		Male		Female		Male		Female		Male		Female	
	13		2		10		3		7		2		11		4	
Age	61.6 ± 6.6				64.2 ± 10.4				61.4 ± 10.5				63.8 ± 8.9			
Height (cm)	165.0 ± 6.8				163.5 ± 7.7				164.0 ± 10.4				160.0 ± 7.1			
Weight (kg)	67.2 ± 10.2				65.2 ± 13.1				65.8 ± 14.3				62.2 ± 13.0			
BMI (kg/m^2^)	24.7 ± 3.6				24.2 ± 3.6				24.1 ± 3.3				24.2 ± 3.3			
Preoperative pain (*n*)	No		Yes		No		Yes		No		Yes		No		Yes	
	12		1		13		2		9		6		9		0	
Preoperative VAS score (average)	1.8				3.2–5.0 (4.1±0.9)				1.0–7.0 (2.7±2.0)				0			
Operative time (min)	139.8 ± 44.6				145.2 ± 45.5				117.7 ± 29.2				127.1 ± 28.7			
No. of ruptured tendons (*n*)	Zero	One	Two	Three	Zero	One	Two	Three	Zero	One	Two	Three	Zero	One	Two	Three
	0	1	10	4	0	2	9	2	0	3	4	2	0	1	11	3
Tenotomy (*n*)	No		Yes		No		Yes		No		Yes		No		Yes	
	6		9		8		5		4		5		6		9	
Interscalene brachial plexus block (*n*)	Without nerve		With nerve		Without nerve		With nerve		Without nerve		With nerve		Without nerve		With nerve	
	9		6		6		7		4		5		5		10	
IV-PCA	No		Yes		No		Yes		No		Yes		No		Yes	
	2		13		4		9		4		5		5		10	
Analgesic use (average) (frequency)	1.6 ± 1.5				1.2 ± 1.6				1.6 ± 1.6				1.7 ± 1.3			
	0–5				0–6				0–4				0–4			

*BMI* body mass index, *IV-PCA* intravenous patient-controlled analgesia, *VAS* visual analogue scale

**Fig. 1:**
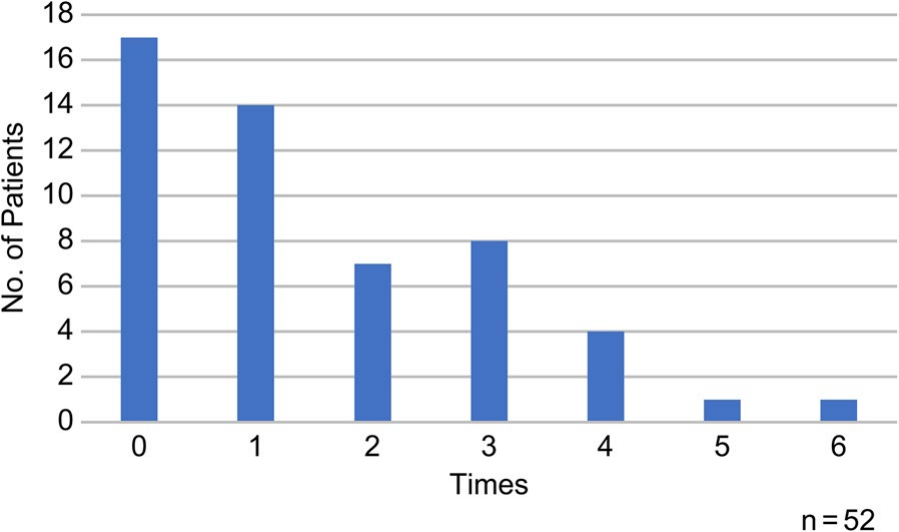
Frequency of analgesic administration for 52 patients. The *y*-axis represents the number of hours after surgery. The *x*-axis represents the number of administrations

Regarding the administration of analgesics for postoperative pain relief, 37 patients (71.2%) received intravenous patient-controlled analgesia (IV-PCA) comprising fentanyl citrate or remifentanil hydrochloride (Table 1) immediately after surgery. Patients were instructed not to tolerate pain. When pain persisted, upon the patient’s wishes, nurses administered analgesics using the following medications: flurbiprofen axetil 50 mg; acelio bag for intravenous injection 1000 mg; pentazocine injection 15 mg and hydroxyzine hydrochloride 25 mg; or diclofenac sodium 25 mg. These analgesics were administered in the aforementioned order. After administration, the reactions of the patients were monitored, and the effect of the analgesic was assessed by asking the patients to rate their pain. When the patients had received analgesics five or six times, they were asked if they wanted a suppository (diclofenac sodium 25 mg). The analgesics were used zero to six times per patient, with a mean frequency of 1.5 times (± 1.5 times) (Fig. 1). Seventeen patients (32.7%) refused analgesic use.

**Fig. 2:**
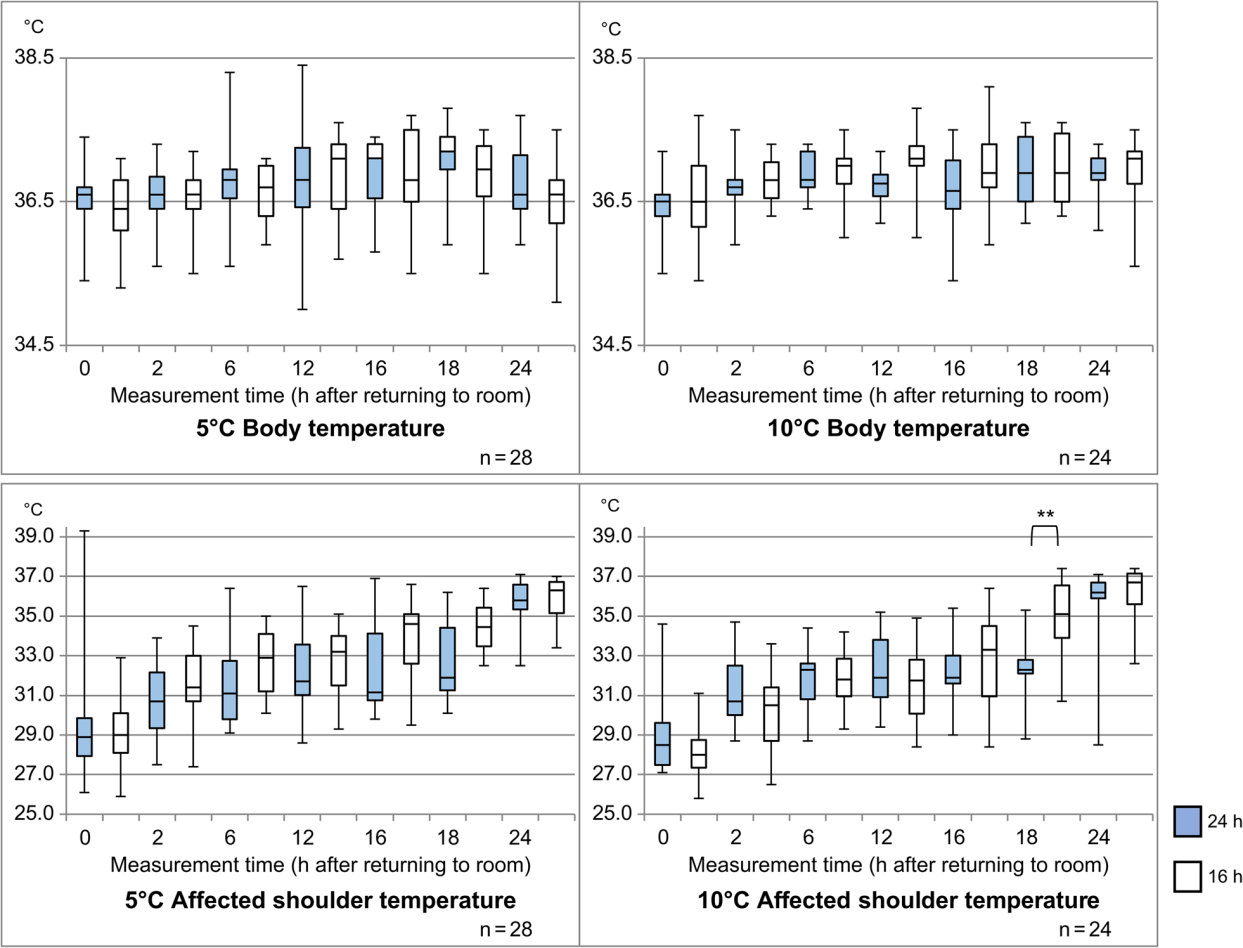
Comparison of body temperatures and affected shoulder temperatures with different durations of cooling temperatures of 5 °C and 10 °C. Significant at **p < 0.01

The body temperature and deep shoulder joint temperature on the affected side are shown in Fig. 2. Body temperatures at both 5 °C and 10 °C indicated no marked fever and no significant differences in the cooling duration. The deep shoulder joint temperature on the healthy side remained 34 °C to 35 °C with cooling temperatures of 5 °C and 10 °C. However, the temperature on the affected side increased to approximately 35.5 °C despite cooling at both 5 °C and 10 °C. During cooling at the different measurement times, the temperature remained in the range of 28 °C to 33 °C; no significant difference was observed between the two cooling durations.

**Fig. 3:**
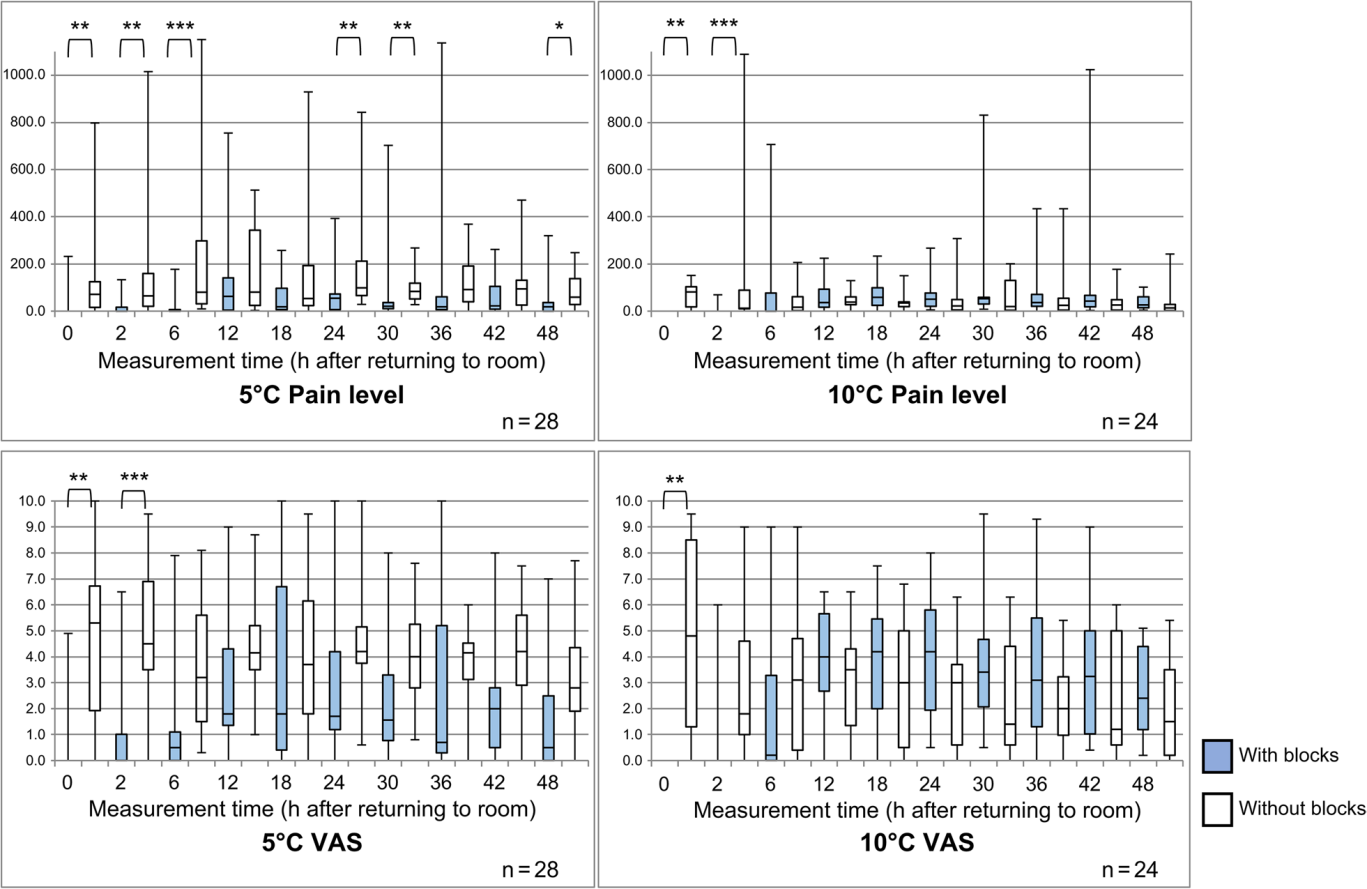
Differences in pain levels and visual analogue scale (VAS) scores with and without anesthesia blocks with cooling temperatures of 5 °C and 10 °C. Significant at *p < 0.05, **p < 0.01, and ***p < 0.001

Since the anesthesia block is considered highly effective for alleviating postoperative pain in affected shoulder joints, the results were compared after first classifying patients according to the presence or absence of an anesthesia block. At 5 °C, 13 patients received an anesthesia block and 15 did not. In terms of the relationship between the use of an anesthesia block and pain, there was a significant difference in VAS scores immediately and up to 2 h after returning to the room (*p* = 0.001 and *p* = 0.0002, respectively). There were significant differences in the pain level immediately, 2 h, and 6 h after returning to the room (*p* = 0.002, *p* = 0.004, and *p* = 0.0002), and at 24, 30, and 48 h after returning to the room (*p* = 0.007, *p* = 0.006, and *p* = 0.045). The pain level was significantly lower (*p* < 0.05) with anesthesia blocks (Fig. 3).

At 10 °C, 15 patients received an anesthesia block and nine did not. Significant differences in the relationship between pain and the use of an anesthesia block were observed only for objective pain up to 2 h after returning to the room and for subjective pain immediately after returning to the room (*p* = 0.0001 and *p* = 0.002, respectively). The presence or absence of the use of an anesthesia block was associated with the relief of postoperative pain in the affected shoulder joint. Tables 2 and 3 show the subjective and objective pain with and without the use of an anesthesia block. The figures in bold indicate where the significant differences occurred in Table 2 to Table 5.

**Table 2:** Comparison of the groups based on time, the presence of an anesthesia block, and pain levels with cooling temperatures of 5 °C and 10 °C

5 °C														
Measurement time (h after returning to room)	24-h group			16-h group			*p*	24-h group			16-h group			*p*
	With blocks *n* = 6			With blocks *n* = 7				Without blocks *n* = 9			Without blocks *n* = 6			
	Median	Min	Max	Median	Min	Max		Median	Min	Max	Median	Min	Max	
0	0	0	232.0	0	0	22.5	0.772	71.1	0	246.5	57.8	0	797.6	0.651
2	0	0	133.2	0	0	19.8	0.871	117.5	0	1015.6	24.5	3.3	194.0	0.245
6	2.3	0	50.6	1.5	0	177.5	1.000	80.2	19.8	1578.8	64.2	9.5	357.1	0.289
12	111.1	0	403.2	36.5	0	755.0	0.647	224.8	17.4	513.0	25.3	2.5	509.5	0.071
18	45.1	0	141.7	8.8	0	257.1	0.943	131.1	0	929.3	23.5	3.0	565.5	0.099
24	55.1	0	73.2	10.7	0	393.0	0.943	100.7	47.3	843.0	88.7	28.6	327.4	0.637
30	11.9	0	51.2	31.8	11.0	702.7	0.109	88.6	50.8	267.9	31.9	27.7	246.4	0.205
36	60.0	0	104.9	6.8	0	1136.8	0.507	96.2	0	368.8	74.3	10.2	209.5	0.372
42	18.0	4.4	174.0	22.4	0	261.8	1.000	127.0	74.4	470.6	16.3	0	121.4	0.007^**^
48	12.4	0	39.7	19.0	0	320.0	0.664	100.7	25.9	247.7	27.4	0	84.5	0.045^*^

10 °C														
Measurement time (h after returning to room)	24-h group			16-h group			*p*	24-h group			16-h group			*p*
	With blocks *n* = 5			With blocks *n* = 10				Without blocks *n* = 4			Without blocks *n* = 5			
	Median	Min	Max	Median	Min	Max		Median	Min	Max	Median	Min	Max	
0	0	0	0.0	0	0	0.0	1.000	100.7	81.3	115.2	18.0	0	151.4	0.140
2	0	0	69.3	0	0	10.9	0.535	21.9	10.0	208.6	12.4	0	1088.7	0.806
6	0.0	0	27.9	5.1	0	706.3	0.161	26.4	1.7	206.4	9.8	0	193.8	0.461
12	23.3	0	79.3	47.9	0	224.3	0.230	81.2	33.1	129.3	34.4	0	66.0	0.355
18	22.2	0	161.2	59.3	23.3	233.3	0.257	37.4	5.4	150.3	26.8	0	120.7	0.327
24	60.0	5.3	267.2	33.5	11.9	203.4	0.462	35.6	18.6	248.3	5.0	0	307.5	0.327
30	46.7	12.8	68.8	53.6	8.1	830.8	0.570	11.6	1.6	200.7	21.2	0	138.6	0.806
36	44.3	15.3	133.0	35.8	0.0	434.3	0.758	24.6	5.3	434.3	21.5	0	93.3	0.564
42	66.7	6.6	366.7	40.2	2.7	1023.7	0.548	23.1	4.2	177.4	44.8	0	155.5	0.806
48	27.3	6.4	101.7	16.1	4.0	65.8	0.386	8.4	1.0	242.2	26.3	0	66.4	1.000

Significant at *p < 0.05 and **p < 0.01

**Table 3:** Comparison of groups based on time, the presence of an anesthesia block, VAS scores, and cooling temperatures of 5 °C and 10 °C

5 °C										
Measurement time (h after returning to room)	24-h group	16-h group	*p*	*t*	Degrees of freedom	24-h group	16-h group	*p*	*t*	Degrees of freedom
	With blocks *n* = 6	With blocks *n* = 7				Without blocks *n* = 9	Without blocks *n* = 6			
0	0.3 ± 0.6	0.8 ± 1.8	0.533	0.644	11	4.1 ± 3.4	5.8 ± 3.9	0.390	0.891	12
2	1.3 ± 2.6	0.7 ± 1.0	0.633	− 0.490	11	5.3 ± 3.0	4.7 ± 2.0	0.719	− 0.368	12
6	0.6 ± 0.5	1.8 ± 2.9	0.292	1.147	6.387	3.9 ± 2.9	3.2 ± 2.2	0.618	− 0.511	13
12	1.9 ± 1.8	4.1 ± 3.5	0.235	1.271	9	5.2 ± 2.4	3.5 ± 1.4	0.159	− 1.5	12
18	2.2 ± 3.3	4.1 ± 3.8	0.360	0.955	11	4.2 ± 3.5	4.0 ± 1.8	0.914	− 0.745	13
24	1.8 ± 1.9	3.8 ± 3.2	0.206	1.346	11	5.1 ± 2.5	3.3 ± 1.2	0.143	− 1.561	13
30	0.9 ± 0.9	3.9 ± 2.7	0.043^*^	2.556	6.047	4.4 ± 2.1	3.4 ± 1.7	0.392	− 0.889	12
36	0.3 ± 0.2	4.1 ± 3.7	0.034^*^	2.718	6.065	3.9 ± 1.9	3.3 ± 1.4	0.540	− 0.635	10
42	1.2 ± 0.9	3.5 ± 2.9	0.089	1.958	7.443	5.0 ± 1.7	2.7 ± 1.7	0.023^*^	− 2.58	13
48	0.6 ± 0.8	2.4 ± 2.6	0.140	1.591	11	2.7 ± 1.4	3.8 ± 2.6	0.321	1.033	13

10 °C										
Measurement time (h after returning to room)	24-h group	16-h group	*p*	*t*	Degrees of freedom	24-h group	16-h group	*p*	*t*	Degrees of freedom
	With blocks *n* = 5	With blocks *n* = 10				Without blocks *n* = 4	Without blocks *n* = 5			
0	0 ± 0	0 ± 0				4.9 ± 3.4	5.0 ± 4.6	0.978	0.029	7
2	1.2 ± 2.7	0.4 ± 1.4	0.465	− 0.752	13	3.5 ± 2.5	2.7 ± 3.8	0.757	− 0.321	7
6	2.0 ± 3.9	1.9 ± 2.4	0.948	− 0.066	12	3.2 ± 2.0	3.2 ± 4.2	0.984	− 0.021	5.995
12	3.9 ± 2.4	3.9 ± 2.2	0.997	− 0.003	12	3.5 ± 1.1	2.9 ± 3.0	0.816	− 0.248	4
18	2.7 ± 2.4	4.4 ± 2.3	0.211	1.315	13	3.8 ± 2.9	2.1 ± 2.3	0.346	− 1.011	7
24	3.6 ± 2.2	4.2 ± 2.5	0.659	0.452	13	3.1 ± 1.6	2.1 ± 2.6	0.524	− 0.671	7
30	2.6 ± 1.6	4.7 ± 2.9	0.179	1.446	10	2.4 ± 2.1	2.4 ± 2.8	0.977	0.030	7
36	3.2 ± 3.0	4.1 ± 2.8	0.579	0.572	11	2.5 ± 1.8	2.2 ± 2.4	0.846	− 0.202	6
42	3.1 ± 1.9	3.7 ± 3.0	0.696	0.400	12	2.5 ± 2.1	2.5 ± 2.8	0.995	0.006	7
48	2.3 ± 1.9	3.0 ± 1.9	0.502	0.693	12	2.1 ± 1.8	2.1 ± 2.3	0.984	0.021	7

Significant at *p < 0.05

### Pain at 5 °C

The pain level with an anesthesia block was not significantly different between groups at all measurement times. The pain level of the 16-h group was significantly higher at 30 h and 36 h (*p* = 0.043 and *p* = 0.034, respectively) after returning to the room according to the comparisons of the VAS scores at different times.

A significant difference in the pain level of the 16-h group was observed between patients with and without anesthesia block at 42 h and 48 h after returning to the room (*p* = 0.007 and *p* = 0.045, respectively). In other words, in the 16-h group, the lowest pain levels were recorded at 42 h and 48 h. Additionally, the 16-h group had significantly lower VAS scores at 42 h after returning to the room than the 24-h group (*p* = 0.023).

### Pain at 10 °C

No significant difference in the pain level at all measurement times was observed for patients with or without an anesthesia block. There was no significant difference in VAS scores between groups at all measurement times with or without an anesthesia block.

The results of the correlation between the pain level and VAS score are shown in Tables 4 and 5. A positive correlation was found for all but one site with an anesthesia block at 5 °C, for all but four sites without an anesthesia block at 5 °C, and for all but one site with an anesthesia block at 10 °C, indicating that the pain levels and VAS scores were similar.

**Table 4:** Correlations between pain levels and VAS scores with an anesthesia block at a cooling temperature of 5 °C

Pain level with blocks *n* = 13											
	**Measurement time (h after returning to room)**	0	2	6	12	18	24	30	36	42	48
**VAS**	0	0.306									
	2		0.766^**^								
	6			0.882^***^							
	12				**0.588**						
	18					0.731^**^					
	24						0.322				
	30							**0.412**			
	36								0.209		
	42									**0.502**	
	48										0.608^*^

Pain level without blocks *n* = 15											
	**Measurement time (h after returning to room)**	0	2	6	12	18	24	30	36	42	48
**VAS**	0	**0.443**									
	2		**0.500**								
	6			0.298							
	12				0.659^*^						
	18					0.573^*^					
	24						0.153				
	30							0.296			
	36								0.698^*^		
	42									**0.502**	
	48										0.099

Significant at *p < 0.05, **p < 0.01, and ***p < 0.001

**Table 5:** Correlations between pain levels and VAS scores with an anesthesia block at a cooling temperature of 10 °C

Pain level with blocks *n* = 15											
	**Measurement time (h after returning to room)**	0	2	6	12	18	24	30	36	42	48
VAS	0	–									
	2		0.883^***^								
	6			0.582^*^							
	12				0.218						
	18					0.804^**^					
	24						**0.443**				
	30							0.770^**^			
	36								0.642^*^		
	42									0.654^*^	
	48										**0.409**

Pain level without blocks *n* = 9											
	**Measurement time (h after returning to room)**	0	2	6	12	18	24	30	36	42	48
**VAS**	0	**0.462**									
	2		0.810^**^								
	6			0.757^*^							
	12				**0.553**						
	18					0.807^**^					
	24						0.826^**^				
	30							**0.566**			
	36								**0.561**		
	42									0.827^**^	
	48										0.677^*^

Significant at *p < 0.05, **p < 0.01, and ***p < 0.001

After 48 h, the retrospective VAS score was obtained by asking the 24-h group to evaluate their pain level during the first 24 h and asking the 16-h group to evaluate their pain level during the first 18 h. Then, the average value was calculated. The same was performed for the VAS scores that had been measured previously. The retrospective VAS score and the measured VAS score were compared (Table 6). There was a significant difference in the retrospective VAS scores of patients in the 24-h group with blocks and without blocks subjected to cooling at 5 °C. Additionally, according to the retrospective VAS scores, patients without blocks experienced stronger pain (7.1 ± 1.5) than those with blocks (4.6 ± 2.9).

**Table 6:** Comparison of measured VAS scores and retrospective VAS scores at cooling temperatures of 5 °C and 10 °C

**5 °C**						
VAS score (average)	**24-h group (average, 24 h)** ***n*** **= 15**			**16-h group (average, 18 h)** ***n*** **= 13**		
	With blocks	Without blocks		With blocks	Without blocks	
	1.3 ± 2.0	4.6 ± 2.9		2.2 ± 3.0	4.3 ± 2.5	
Retrospective VAS score	**24-h group *n*** **= 15**		***p***	**16-h group** ***n ***= **13**		***p***
	With blocks	Without blocks		With blocks	Without blocks	
	2.5 ± 2.5	7.1 ± 1.5	0.005*	4.6 ± 4.0	5.5 ± 3.1	0.653

**10 °C**						
VAS score (average)	**24-h group (average, 24 h)** ***n*** **= 9**			**16-h group (average, 18 h)** ***n*** **= 13**		
	With blocks	Without blocks		With blocks	Without blocks	
	2.2 ± 2.7	3.7 ± 2.3		2.1 ± 2.5	3.2 ± 3.5	
Retrospective VAS score	**24-h group** ***n*** **= 9**		***p***	**16-h group** ***n*** **= 13**		***p***
	With blocks	Without blocks		With blocks	Without blocks	
	4.5 ± 3.2	4.3 ± 3.8	0.95	6.1 ± 2.8	2.6 ± 4.2	0.147

Significant at *p < 0.01

**Fig. 4:**
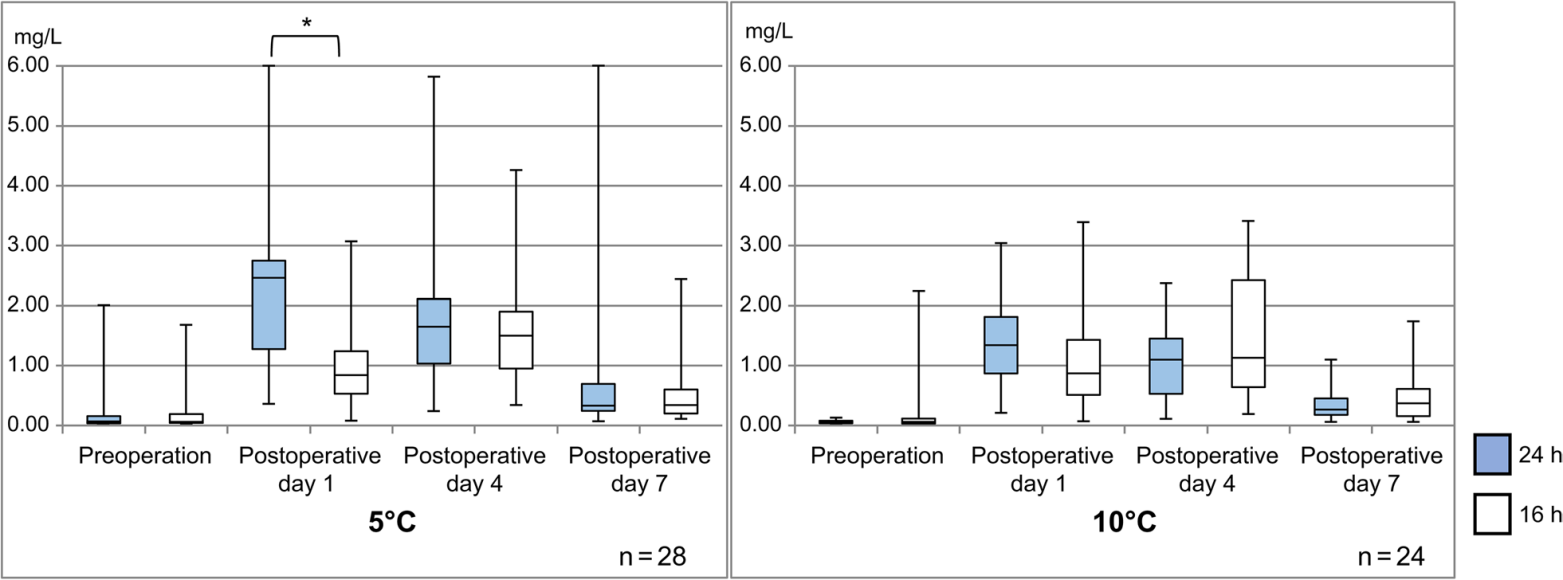
Comparison of measured C-reactive protein (CRP) levels with cooling temperatures of 5 °C and 10 °C. Significant at *p < 0.05

The CRP level at 5 °C was highest in the 24-h group (2.56 ± 1.91 mg/dL on postoperative day 1) and in the 16-h group (1.84 ± 1.18 mg/dL on postoperative day 4). A comparison based on the cooling time showed that the CRP level was significantly higher in the 24-h group (*p* = 0.015) only on the postoperative day 1. Similarly, the CRP level at 10 °C was highest in the 24-h group on postoperative day 1 (1.37 ± 0.92 mg/dL) and in the 16-h group on postoperative day 4 (1.46 ± 1.07 mg/dL); however, there was no significant difference between groups (Fig. 4). Inflammation usually decreases over time after surgery. Blood samples for CRP measurements on postoperative day 1 were obtained at 18 h after surgery for the 24-h group and at 13 to 14 h after surgery for the 16-h group. However, even though blood collection was performed 18 h postoperatively, the CRP level on the postoperative day 1 showed a stronger inflammatory response in the 24-h group. Therefore, the CRP levels were compared according to the operative times, the number of tendons that underwent surgery, and tenotomy, which are related to the inflammatory response. The results showed that the CRP level was significantly higher in the 24-h group (*p* < 0.05), which had a longer operative time, more tendons that underwent surgery, and a higher rate of tenotomy.

No adverse events, such as temperature allergy, frostbite, discomfort from cooling, prolonged healing of surgical wounds, or sensory disturbance, were observed during cryotherapy in this study. Therefore, discontinuation of cooling therapy because of adverse events was not required.

## Discussion

Shoulder rotator cuff injuries, which are more common in men older than 40 years of age and have a peak age of onset of approximately 60 years, are the primary reasons for arthroscopic shoulder surgery. Although the proportion of men in this study was slightly higher, a similar trend was observed. The basic characteristics of our study participants broadly agree with the characteristics of disease onset.

The temperature in the deep shoulder joint increased to almost the same temperature as that of the healthy side even with cooling after 24 h of surgery. At the end of cooling, in the 16-h group at 10 °C, the increase to the same temperature in the deep shoulder joint as that of the healthy side was observed. However, no difference in the temperature of the joint during cooling was caused by cooling temperatures of 5 °C or 10 °C. The results showed that there was no difference in the temperature within the joint during cooling at 5 °C and 10 °C.

Inflammation after shoulder arthroplasty has been reported [33–38] to be more intense with longer durations of surgery, more operated tendons, and more tendon dissection. Regarding the relationships among the CRP levels and the duration of surgery, number of operated tendons, and tendon dissection, our study also showed higher CRP levels and stronger inflammatory responses with longer surgery times, more operated tendons, and more tendon dissection. Therefore, although the present study confirmed that inflammation was significantly related to the operative time and procedure, the postoperative inflammatory response of CRP levels did not reveal a cryotherapy effect.

Pain after arthroscopic shoulder surgery is related to the presence or absence of a surgical anesthesia block; in the absence of a block, pain is present immediately after returning to the room (depending on the individual). To obtain effective analgesia with cryotherapy, it is necessary to vary the cooling temperature and duration of cryotherapy based on the presence or absence of the anesthesia block.

At 5 °C, with an anesthesia block, the objective pain level was not related to the cooling time. However, there was a significant difference in subjective pain levels. The 16-h group experienced more pain at 1.5 days after surgery. Therefore, it is suggested that continuous cooling at 5 °C for 24 h is better for reducing sensory pain.

However, without an anesthesia block, the 16-h group reported less pain overall and at two days postoperatively. The 24-h group reported significantly more objective and subjective pain, suggesting that 16 h is the optimal cooling time without an anesthesia block.

The VAS is a universal subjective pain assessment tool used in various fields. However, the PAINVISON was created in Japan to assess objective pain. Although it is currently being introduced into medical practice, its usefulness is still being studied. Therefore, in this study, the VAS was considered essential.

At 10 °C, there was no difference in cooling times in terms of both objective and subjective pain with or without an anesthesia block. However, the mean VAS scores were higher in the 16-h group at 18 h after surgery. Additionally, the retrospective VAS scores, which was performed within one or two days after the end of cryotherapy, were not different between the 24-h group with and without block; however, the 16-h group with an anesthesia block felt more pain (approximately 4 cm more according to the VAS) during cryotherapy. Additionally, the 10 °C and 16-h group experienced an increase in deep shoulder joint temperature when continuous cooling was discontinued. Therefore, at 10 °C, objective and sensory pain were not affected by 24 h and 16 h of continuous cooling. However, in the presence of an anesthesia block, it was suggested that, whenever possible, 24 h of cooling at 10 °C was preferable to 16 h of cooling to reduce sensory pain.

This study was limited because it was difficult to include the use of analgesics in the analysis. Because the patients were advised not to tolerate pain, they were administered analgesics as soon as they experienced any pain. In addition, different types of analgesics were administered prophylactically before the pain intensified. Furthermore, if the use of analgesics had been included in the analysis, then there would have been more variables. The number of patients would have become statistically insufficient for inter-group comparisons. Therefore, we were unable to examine the effect of analgesics on the studied parameters.

## Conclusions

Cryotherapy was investigated to determine its benefits in terms of pain relief. Pain after arthroscopic shoulder surgery was related to the presence or absence of a brachial plexus block using the interscalene approach during surgical anesthesia. It was necessary to change the postoperative cooling temperature and time depending on the presence or absence of the brachial plexus block using the interscalene approach. We propose that the cryotherapy cooling temperature range should be 5 °C to 10 °C; however, 5 °C for 24 h appears to be optimal for patients with an anesthesia block. For patients without an anesthesia block, cooling at 5 °C for 16 h and at 10 °C for 24 h provides optimal benefits. The participants of this study were patients with rotator cuff injuries. Hence, in the future, we intend to investigate further applications of cryotherapy and compare its efficacy for treating patients who underwent arthroscopic shoulder surgery because of other disorders.

Importantly, our findings support the view that universal cryotherapy intensity and duration settings regardless of the use of other interventions will unintentionally increase postoperative pain levels. Therefore, the results of this study could provide a guide for determining the temperature and duration of cryotherapy after arthroscopic shoulder surgery.

## Data Availability

The data generated in this study have not been published. Therefore, the data are not available elsewhere. In addition, the data have been statistically processed to ensure that individual privacy is not violated.

## Abbreviations

VAS: Visual analog scale
CRP: C-reactive protein
